# Coloring and Strength Effects of Calcined Manganese Ore in Ceramic Brick Containing Ash-Slag Waste

**DOI:** 10.3390/ma19204313

**Published:** 2026-10-13

**Authors:** Ruslan Safarov, Aigerim Takirova, Askhat Rakhimov, Aliya Aldungarova, Ayat Sabitov, Yerlan Baikenov, Ruslan Kamatov, Zhanat Shomanova

**Affiliations:** 1 Department of Chemistry, Institute of Natural Sciences, L.N. Gumilyov Eurasian National University, Astana 010008, Kazakhstan; safarov_rz@enu.kz (R.S.); sabitov_as_3@enu.kz (A.S.); baikenov_yea_1@enu.kz (Y.B.); 2 Department of Chemistry and Chemical Technology, Kh. Dosmukhamedov Atyrau University, Atyrau 060011, Kazakhstan; 3 Department of Building Materials and Technologies, Abylkas Saginov Karaganda Technical University, Karaganda 100027, Kazakhstan; 4 School of Engineering, International Educational Corporation, Almaty 050043, Kazakhstan; liya1479@gmail.com; 5 Department of Science, L.N. Gumilyov Eurasian National University, Astana 010008, Kazakhstan; kamatov_rm@enu.kz; 6 Higher School of Natural Science, Margulan University, Pavlodar 140002, Kazakhstan; zshoman@yandex.ru

**Keywords:** hausmannite, Mn_3_O_4_, calcined manganese ore, fired clay brick, coal ash valorization, Ekibastuz coal ash, compressive strength, CIELAB colorimetry, decorative ceramics

## Abstract

**Highlights:**

- Adding 7 wt.% calcined Bogach manganese ore to a brick body of clay and ash-slag waste raised the mean compressive strength from 9.89 to 12.41 MPa after firing at 950 °C; the ore-free body with ash-slag waste does not reach grade M100, whereas the ore-containing one does.
- Crystalline hausmannite (Mn_3_O_4_) introduced with the calcined ore persisted after firing at 950 °C in both ore-containing bodies, with and without ash-slag waste, and was absent from the ore-free bodies.
- The additive partly offsets the strength loss caused by replacing 20 wt.% of the clay with ash-slag waste; with three specimens per mix, the strength gain is a tendency that requires confirmation on a larger sample.
- In the body containing ash-slag waste, the same additive lowers the lightness L* from 67.3 to 43.3 (ΔE_00_ = 22.9 relative to the ore-free body) at an essentially constant hue angle; the body is colored during the same firing, without a separate decoration step.

**Abstract:**

The replacement of clay by coal ash in fired brick is limited by the resulting strength loss. This study examines whether a calcined manganese ore can color a brick body containing ash-slag waste (ASW) without reducing its strength. Four mixes of a medium-plasticity clay with 0 or 20 wt.% ASW (Pavlodar thermal power plant TPP-1) and 0 or 7 wt.% Bogach manganese ore calcined at 1200 °C were fired at 950 °C and characterized by X-ray fluorescence, X-ray diffraction with Rietveld refinement, image-based CIELAB colorimetry and physico-mechanical tests. In the ASW-containing body, the ore raised the mean compressive strength from 9.89 ± 1.33 to 12.41 ± 3.46 MPa (three specimens per mix, *p* = 0.34) without significant changes in water absorption and density; by mean strength, this body corresponds to grade M100 (GOST 530-2012), class f_b_ ≥ 10 MPa (EN 771-1) and grade NW (ASTM C62-17). Hausmannite (Mn_3_O_4_) introduced with the ore persisted as a crystalline phase after firing in both ore-containing mixes (about 2% of the crystalline fraction); the mechanism of the strength change was not established. The ore lowered the lightness L* of the ASW-containing body from 67.3 to 43.3 (ΔE_00_ = 22.9) at an essentially constant hue angle (57–60°); the fired body is dark brown.

## 1. Introduction

Construction ceramics carry a high specific CO_2_ burden: the production of fired clay brick generates substantial direct and indirect emissions associated with raw-material extraction, firing and logistics [1]. As climate commitments tighten and circular-economy principles are incorporated into legislation, the reuse of waste materials in construction products has acquired both a regulatory and an economic rationale [2,3]. In Kazakhstan this agenda is reinforced by the scale of the waste streams: about 19 million tonnes of coal ash are generated annually, more than 300 million tonnes have accumulated in ash disposal areas, and only about 10% of the ash is utilized [4,5]; the ash and slag stored in these areas are referred to below as ash-slag waste (ASW). In parallel, the central regions of the country hold explored reserves of manganese ore that are traditionally directed to metallurgical processing [6]; extending their use to functional construction materials is consistent with the broader effort to diversify the regional mineral-resource base [7]. Combining an industrial ASW stream with a natural manganese feedstock within a single ceramic technology could therefore use both resources in one product and reduce the environmental footprint of construction-ceramic production.

The use of coal ash in fired ceramics has been examined systematically for both Class F and Class C ashes as defined by ASTM C618 [8,9]. Up to about 20% of the clay can typically be replaced with coal ash while meeting common strength requirements, provided that the firing temperature is sufficiently high, whereas higher ash shares lower the compressive strength and increase the water absorption as open porosity develops [8,9,10]; coal ashes are also used in unfired, cement-bound bricks [11]. Three concurrent factors account for the strength loss: dilution of the ceramic matrix by the glassy phase and quartz supplied with the ash, burnout of residual carbon and organic matter with the formation of open porosity [12], and incomplete liquid-phase sintering at moderate firing temperatures [9,10,13]. The ash of the Ekibastuz coal basin, used in the power plants of northern and central Kazakhstan, is a Class F ash with a high combined content of SiO_2_, Al_2_O_3_ and Fe_2_O_3_ and a low CaO content (for example, 90% and 6%, respectively, for an Ekibastuz fly ash [4]), and its mineral fraction comprises α-quartz, mullite and an amorphous aluminosilicate glassy phase [4,14,15]. Additional industrial components, such as waste glass and slags, can partially compensate for the strength loss by promoting liquid-phase sintering [14,16,17]. To date, no systematic data have been published on the combination of Ekibastuz coal ash with manganese ore in fired ceramics.

Manganese compounds have long been used to color ceramics and glass, yielding tones from lilac to dark brown depending on the Mn oxidation state and the character of the matrix [18,19,20]. Recent work has revived interest in these effects in the context of mining-waste reuse, where the phase composition [21], strength and color [22,23] of brick bodies containing manganese ores, beneficiation tailings, manganese-containing wastes and pigments have been examined. Manganese-containing wastes have been reported to color brick bodies in bulk without lowering their strength, although oxide pigments in general can disturb the sintering of the clay minerals [23]. In such bodies, the decorative effect has been attributed to the high absorption coefficient of hausmannite (Mn_3_O_4_) in the visible range, which originates from ligand-to-metal charge-transfer (LMCT) transitions O^2−^ → Mn^3+^ in the 410–585 nm region together with weak d–d transitions near 810 nm [24]. Electrolytic manganese residue and fly ash have recently been combined in engineered ceramic composites [25], which shows that two industrial streams can be used in one ceramic product. The transfer of comparable schemes to the natural manganese ores of Central Kazakhstan has not been reported.

Among the manganese oxides, Mn_3_O_4_ (hausmannite) is the thermodynamically stable phase in air above approximately 890 °C, as follows from thermodynamic data and CALPHAD (calculation of phase diagrams) assessments of the Mn–O system [26,27] and from calcination studies of manganese ores [28]. It crystallizes in the tetragonal structure with space group I4_1_/amd (a = b = 5.769 Å, c = 9.461 Å for synthetic α-Mn_3_O_4_ [29]). At about 1170 °C, tetragonal Mn_3_O_4_ transforms in air into a cubic spinel [27,30]. On heating the higher oxides (MnO_2_, Mn_2_O_3_) in an oxidizing atmosphere, stepwise reduction proceeds towards Mn_3_O_4_ [7,26,31]. The redox behavior of Mn_3_O_4_ depends on crystallite size: nanocrystalline Mn_3_O_4_ (tens of nanometers) has a larger specific surface area and size-dependent oxidation–reduction kinetics [32,33,34], which may affect its interaction with the ceramic matrix, while its intense dark-brown color arises from electronic transitions in the visible range [24]. This combination of properties makes Mn_3_O_4_ a candidate for fulfilling strengthening and decorative functions in a ceramic body at the same time.

Separate studies exist on the use of Ekibastuz ash and on the manganese ores of Central Kazakhstan, but systematic data on their joint introduction into a fired ceramic batch are lacking. In particular, the phase-formation mechanisms in the ternary system clay–ASW–manganese ore at standard brick-firing temperatures have not been described, the contribution of a pre-formed hausmannite phase to the physico-mechanical properties of the ceramic body has not been studied, and the resulting bodies have not been benchmarked simultaneously against the regional, European and American brick standards.

The present study therefore evaluates the effect of a manganese-ore addition from the Bogach deposit on the physico-mechanical properties, phase composition and decorative characteristics of ceramic brick based on ash-slag waste from the Pavlodar thermal power plant (TPP-1). The practical aim was to obtain a dark-colored brick body in which one fifth of the clay is replaced by ASW while the lowest strength grades of the regional, European and American standards are still met. The work was undertaken because the two feedstocks are available in large volumes in the same region yet have not previously been combined in a fired ceramic body. Four mixes were prepared with variable ASW content (0 or 20% of the clay–ASW blend) and manganese-ore addition (0 or 7% of the total batch mass, introduced as ore calcined at 1200 °C) and fired at 950 °C. They were characterized by X-ray fluorescence analysis (XRF), by X-ray diffraction (XRD) with Rietveld phase refinement, a method widely used to follow the mineralogical changes of clay bodies on firing [35,36], by CIELAB colorimetry using the CIEDE2000 formula [37], and by compressive-strength, water-absorption, linear-shrinkage and mass-loss tests. The novelty of the work lies in the combined use of an ash-slag waste and a calcined manganese ore containing a pre-formed hausmannite phase in a fired ceramic body, in the characterization of the phase composition, strength and color of the resulting bodies, and in benchmarking them against three regulatory systems.

## 2. Materials and Methods

### 2.1. Raw Materials

The plasticity of the clay was determined according to [38] (liquid limit by the balancing-cone method): liquid limit W_L_ = 33.5%, plastic limit W_P_ = 17.0% and plasticity index PI = 16.5%. According to the classification of clay raw materials for the ceramic industry [39], in which the plasticity index of medium-plasticity clays is 15–25, the raw material belongs to the medium-plasticity clays.

The oxide composition of the clay, determined by XRF on a fired specimen (Mix 1) and normalized to 100%, is reported together with the consolidated data for all ceramic mixes in Section 3.1. The composition is dominated by SiO_2_ (62.03%) and Al_2_O_3_ (17.89%). According to XRD (Section 3.2.3), the clay minerals are represented by a dioctahedral smectite of the montmorillonite type, muscovite/illite and clinochlore, accompanied by quartz, cristobalite, albite and calcite, with dolomite, a heulandite-type zeolite, K-feldspar and anatase at trace level; kaolinite was not confirmed. The Fe_2_O_3_ content (7.54%) accounts for the red-firing behavior of the raw material, and the total content of fluxing oxides (CaO + MgO + K_2_O ≈ 9.3%) can promote the formation of a liquid phase at the firing temperature of 950 °C.

A sample of the ash-slag waste was taken from the ash disposal area of Pavlodar TPP-1 (Aluminium of Kazakhstan JSC (joint-stock company), Pavlodar, Kazakhstan), where the combustion products of Ekibastuz coal are stored. Its chemical composition, determined by X-ray fluorescence and recalculated from the elemental to the oxide form (Table 1), is dominated by silicon dioxide with a substantial alumina content. The high concentration of aluminosilicate components, combined with the presence of potassium, calcium and magnesium oxides, can promote the formation of a liquid phase during thermal treatment.

According to the particle-size analysis (Table 2), the ash-slag waste is a fine-grained material: the cumulative content of fractions below 0.315 mm is 88.3%, and the largest fraction (46.5%) is the 0.315–0.16 mm range.

Manganese ore from the Bogach deposit (Nurinsky district, Karaganda region, Kazakhstan) was used as a color modifier. In the as-supplied state the ore is a dark, almost black lump rock. It was crushed to 1–5 mm in a jaw crusher, ground in a laboratory ball mill for 8 h and sieved to a particle size of not more than 0.075 mm. The powder was calcined in a corundum crucible in a muffle furnace at 1200 °C for 4 h in air and cooled together with the furnace, and the sintered product was ground in a porcelain mortar without subsequent sieving. The elemental composition of the ore in the as-received state and after calcination at 1200 °C is given in Table 3. The XRF values were obtained by the standardless method and are given without normalization to 100% (O, C and H are not determined; the sums of the determined elements are 78.3% and 80.0%). When recalculated to oxides with Mn as Mn_3_O_4_, the sum for the calcined ore is about 113%, which indicates that the standardless analysis overestimates the absolute contents in this Mn-rich material. The values are therefore used here to compare the two states, and the Mn contents (60.77% and 65.24%) should be regarded as upper estimates.

The mineralogical composition of the as-received ore is a complex assemblage of oxides, silicates and carbonates dominated by cryptomelane, braunite, calcite and quartz, with trace hollandite [7]. The braunite–calcite–quartz part of this assemblage is typical of the Atasu-type manganese ores of Central Kazakhstan, as shown for the Ushkatyn-III deposit [40], whereas cryptomelane and hollandite are supergene manganese oxides [18].

### 2.2. Batch Compositions and Specimen Preparation

The study was carried out on four mixes (Table 4). For each formulation, five to six cylindrical specimens were formed (diameter 48–51 mm and height 45–53 mm in the green state). One specimen of Mix 1 and one of Mix 2 had heights of 58 and 35 mm, respectively; neither was used for compressive-strength testing. Bulk density, linear shrinkage and mass loss were determined on five to six specimens per mix (Section 3.3), and water absorption and compressive strength on three specimens per mix.

For Mix 4, the batch consisted of 93% of a clay:ASW blend in the 80:20 ratio and 7% of calcined manganese ore, both referred to the total mass of the mixture, which corresponds to 74.4% clay, 18.6% ASW and 7.0% ore (Table 4); Mix 2 was prepared in the same way from 93% clay and 7% ore. The components were mixed in the dry state until homogeneous, and the specimens were formed by plastic molding at a moisture content of 21.4–21.8% for Mixes 2–4 and 19.3% for Mix 1 (calculated on the dry mass). After demolding, the specimens were kept for 24 h under laboratory conditions and dried in an oven at 105 °C to constant mass. Firing was carried out at 950 °C in an electric chamber kiln for ceramics equipped with a programmable 518P controller and a thermocouple (Hebei Zhaoxin Sporting Goods Manufacturing Co., Ltd., Yanshan, Hebei, China), with a heating time of about 2 h, a dwell of 30 min and cooling inside the closed kiln. All mixes were fired in the same kiln using the same program, in separate loads.

To assess the processing behavior, shrinkage indicators were calculated. The mass loss on drying, W_dry_, and the total mass loss after firing, W_fired_ (both referred to the green mass), were obtained from Equations (1) and (2), and the linear shrinkage by diameter, L_d_, and by height, L_h_, from Equations (3) and (4):

W_dry_ = (M_green_ − M_dried_)/M_green_ × 100%, (1)

W_fired_ = (M_green_ − M_fired_)/M_green_ × 100%, (2)

L_d_ = (D_green_ − D_fired_)/D_green_ × 100%, (3)

L_h_ = (H_green_ − H_fired_)/H_green_ × 100%, (4)

where M, D and H denote the mass, diameter and height of the specimen in the green, dried and fired states, as indicated by the subscripts.

### 2.3. Chemical and Mineralogical Characterization

The elemental composition of the raw materials and of the fired mixes was determined by X-ray fluorescence analysis (XRF) on a Bruker S2 PUMA energy-dispersive spectrometer (Bruker AXS, Karlsruhe, Germany) equipped with an X-ray tube with a silver (Ag) anode operated at 20–50 kV and 100–1000 µA with automatic filter selection. Samples were prepared as two-layer pellets on a boric acid backing: the backing was pressed at 5 t, and then pressed together with the sample at 25 t for 3–5 min, following the general recommendations of [41] for pellet preparation. Concentrations were computed with the built-in factory calibration of the instrument software by the standardless fundamental-parameters method with peak-overlap correction; no calibration against matrix-matched certified reference materials was performed. This approach corresponds to the semi-quantitative screening with a matrix-independent calibration set up by the manufacturer (Annex A of [41]) rather than to the main procedure of this standard. The relative standard deviation (RSD) for the major components did not exceed 0.6% (*n* = 9).

The qualitative and quantitative phase composition was determined by X-ray diffraction (XRD) on a Bruker D6 PHASER diffractometer (Bruker AXS, Karlsruhe, Germany) in Bragg–Brentano geometry using Cu Kα radiation (λ = 1.5406 Å) and an SSD160-2 detector. Samples were ground in an agate mortar to a homogeneous powder before measurement; for the fired mixes, fragments of the same cylindrical specimens fired at 950 °C were used. All patterns were recorded at 40 kV and 15 mA with a counting time of 0.8 s per step: the calcined ore over 2θ = 15–85° with a step of 0.02°, the ASW over 2θ = 10–86.5° with a step of 0.055°, and the clay and the fired mixes over 2θ = 10–80° with a step of 0.02°. Candidate phases were selected by search-match against the International Centre for Diffraction Data (ICDD) Powder Diffraction File (PDF-4+, 2025) and the Crystallography Open Database (COD, revision 240108) [42] and verified by Rietveld refinement in Profex 5.4.0/BGMN [43] with the instrument function of the diffractometer and structure models from the Profex/BGMN library and the COD. The lattice parameters were allowed to vary within ±0.3% of the reference values for phases of fixed composition, ±0.5% for solid solutions and ±1% for layer silicates and zeolites. Phases whose refined content did not exceed twice their estimated standard deviation were removed step by step. A phase was accepted only when adding it to the model visibly improved the fit at its characteristic reflections; a phase that improved the fit only at a single overlapped reflection was reported as a trace or rejected. The 2θ scale of each pattern was corrected for sample displacement using the quartz reflections as an internal reference. No internal standard was added to the clay, the ASW or the fired mixes, so the refined contents refer to the crystalline fraction only, and the phase composition of these materials is reported qualitatively (Section 3.2); the only exceptions are the manganese oxides (hausmannite and bixbyite), whose contents are given in wt.% of the crystalline fraction with the estimated standard deviations of the refinement.

### 2.4. Physical and Mechanical Testing

The bulk density of the fired specimens was determined in accordance with [44] as the ratio of the specimen mass to its geometric volume, calculated from the measured diameter and height, using Equation (5):

ρ = 4m/(πd^2^h), (5)

where m is the mass of the fired specimen (g), and d and h are its diameter and height (cm), measured to the nearest 1 mm; the result was converted to kg/m^3^.

Water absorption was determined following [44], except that the specimens were not dried before saturation (see below). The specimens were fully immersed in water at 25 °C, with the water level 5–10 cm above their surface, and kept for 48 h. After saturation, they were removed from the water, the surface water was wiped off with a damp, wrung-out cloth, and the specimens were weighed within 2 min. Water absorption WA was calculated from Equation (6):

WA = (m_sat_ − m_0_)/m_0_ × 100%, (6)

where m_sat_ is the mass of the saturated specimen and m_0_ is the mass of the fired specimen weighed after cooling to room temperature, before immersion; no additional drying was performed, since the specimens were dry after firing. The specimen masses were recorded with a step of 2 g, which corresponds to 1.2–1.7 percentage points of water absorption for specimens of 116–170 g; mean values are tabulated to 0.1%, but differences between mixes smaller than about 1.5 percentage points are not resolved, and all statistics were computed from the unrounded values. According to [45], the water absorption of brick other than clinker brick shall not be less than 6.0%.

Compressive strength was determined on a Controls compression testing machine (Controls S.p.A., Liscate, Italy; calibrated in November 2025) according to [46] (analogous to [47]). The bearing surfaces of the specimens were ground to ensure uniform load distribution, the loading rate was controlled automatically by the machine, and the strength was calculated as the ratio of the failure load to the cross-sectional area of the specimen. The specimens were assigned to strength grades according to [45], in which brick grades M100–M300 correspond to mean compressive strengths from 10 to 30 MPa. Because the flexural strength was not determined and three specimens per mix were tested instead of the five required by [45], the grades given in Section 3.4.1 are estimates based on compressive strength only.

The linear drying and firing shrinkage was determined as the change in the dimensions of the specimen by diameter and by height, and the mass loss as the change in its mass on drying and firing.

### 2.5. Color Characterization

Color characteristics of the fired specimens (one specimen per mix) were quantified from a single digital photograph taken against a white-paper background; the color coordinates in the CIELAB system (CIE 1976 L*a*b*, illuminant D65) were computed with an in-house Python 3.13 script from the JPEG file (the camera recorded RAW and JPEG files simultaneously), assuming the sRGB color space with the D65 white point. The image was normalized to the white-paper region, which served as the white reference and reduced the influence of the illumination conditions on the measured values. A calibrated color chart was not used, so the coordinates are image-based estimates that serve for comparing the mixes rather than as absolute colorimetric values. Mean L*, a* and b* coordinates were determined over the central area of each specimen (80% of the surface); chroma C* = (a*^2^ + b*^2^)^1/2^ and hue angle h° = arctan(b*/a*) were derived from them. The total color difference between each mix and the clay-only control (Mix 1) was calculated using the ΔE_00_ (CIEDE2000) formula [37]; the same formula was used for Mix 4 relative to Mix 3, and the CIE76 difference ΔE*_ab_ was also computed for Mix 4 relative to the control (Section 3.6).

### 2.6. Statistical Analysis

Bulk density, linear shrinkage and mass loss were determined on five to six specimens per mix, and water absorption and compressive strength on three specimens per mix. The results are reported as the arithmetic mean ± standard deviation (x̅ ± s). Differences between pairs of mixes (Mix 3 versus Mix 4 and Mix 1 versus Mix 2) were assessed by Welch’s *t*-test, and the interaction between the ASW and ore factors was tested by two-way analysis of variance of the individual strength values. Relationships between batch composition and the properties of the fired body were quantified by Pearson correlation coefficients (*r*) with the corresponding significance levels (*p*); the correlation analysis was performed over the four between-mix mean values (two degrees of freedom), and *p* < 0.05 was adopted as the threshold of formal statistical significance. All calculations were performed with in-house scripts in Python 3.13 using NumPy 2.2 and SciPy 1.16. The XRD patterns, property plots and the correlation matrix were plotted from the measured data with Matplotlib 3.10 (the correlation matrix with seaborn 0.13), and the specimen photographs were assembled with Pillow 12.3.

## 3. Results

### 3.1. Oxide Composition of the Ceramic Mixes

The XRF results for the oxide composition of all four mixes, including the clay-only control (Mix 1), are presented in Table 5; the analyses were performed on fired specimens. The most discriminating compositional feature is the MnO concentration. In the ore-free mixes (Mixes 1 and 3) it does not exceed 0.14%, whereas the introduction of 7% manganese ore (Mixes 2 and 4) raises MnO to 3.87–4.02%, which confirms the presence of the manganese component. Since the clay-based matrix loses 4.9–6.1% of its dry mass on firing (Section 3.3) and the calcined ore does not, the ore accounts for about 7.3–7.4% of the fired mass. The MnO contents of Mixes 2 and 4 therefore correspond to about 40–42% Mn in the calcined ore, which is notably lower than the value in Table 3 (65.24%) and is consistent with the overestimation of the absolute Mn content by the standardless analysis (Section 2.1). In the ASW-containing mixes, Al_2_O_3_ rises from 17.89% to 19.00% (Mix 3) and 18.71% (Mix 4), and Fe_2_O_3_ reaches 9.06% in Mix 3. Mixing 80% clay with 20% ASW (Al_2_O_3_ 18.6%, Fe_2_O_3_ 7.6%, Table 1) would give only about 18.0% Al_2_O_3_ and 7.6% Fe_2_O_3_ (Table 1 and Table 5), so both deviations exceed the analytical precision and are attributed to compositional heterogeneity of the fired specimens rather than to the ash. The decrease in SiO_2_ in the ore-containing mixes is consistent with the low silicon content of the calcined manganese ore (2.75% Si, Table 3), which dilutes the overall silica background of the system.

### 3.2. Phase Composition of the Raw Materials and Fired Bodies

#### 3.2.1. Phase Evolution of the Bogach Manganese Ore on Calcination

**Figure 1:**
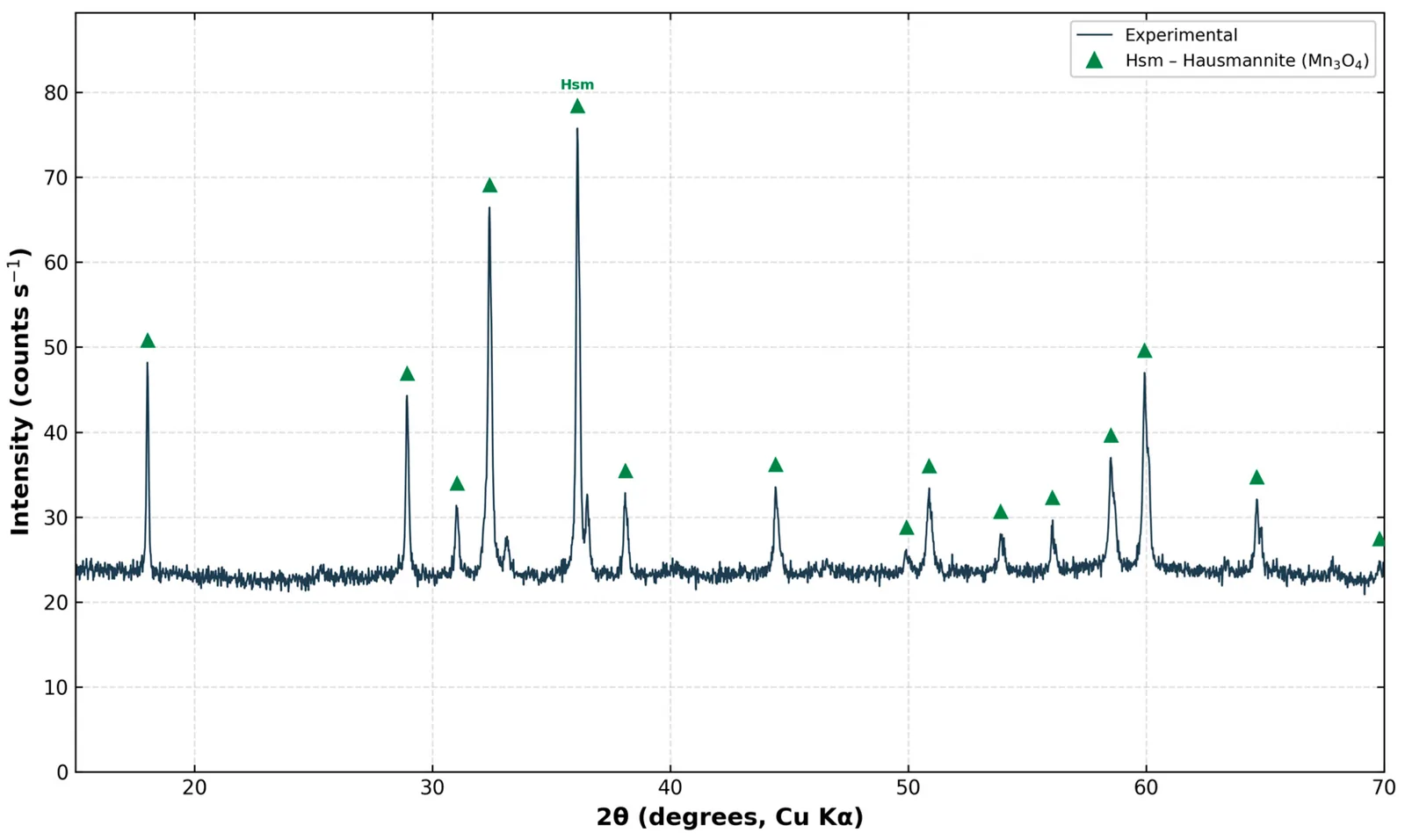
XRD pattern of the manganese ore from the Bogach deposit after calcination at 1200 °C. Hsm—hausmannite (Mn_3_O_4_).

XRD analysis of the Bogach ore after calcination at 1200 °C for 4 h (Figure 1) shows that the original manganese-bearing minerals have transformed. The dominant crystalline phase in the calcined ore is hausmannite (Mn_3_O_4_), identified using COD entry 1514121. The diffraction pattern displays sharp reflections corresponding to the tetragonal hausmannite lattice (space group I4_1_/amd) with refined lattice parameters a = b = 5.763 Å and c = 9.439 Å, slightly smaller than those of synthetic α-Mn_3_O_4_ (a = 5.769 Å, c = 9.461 Å [29]), which may reflect minor cation substitution in the natural material. According to the previously published characterization of the same ore [7], Rietveld refinement gave a hausmannite content of 95.77% of the crystalline phase mass and an average coherent-scattering domain size of 113.4 ± 4.5 nm, and the degree of crystallinity (DOC) determined by the internal-standard method (α-Al_2_O_3_, 15 wt.%) is 25.8%. In the present refinement of the calcined ore, the remaining 4% of the crystalline fraction is described equally well by hematite and quartz (3.4 and 0.8 wt.%) or by braunite (2.8 ± 0.3 wt.%) and bixbyite (1.7 ± 0.5 wt.%), because the strongest reflections of hematite, braunite and bixbyite overlap near 33° (weighted-profile R-factor R_wp_ = 2.77% and 2.73%, respectively). The DOC value may be affected by microabsorption: manganese-bearing phases strongly absorb Cu Kα radiation and produce fluorescence, so that without a microabsorption correction their fraction relative to the corundum standard is underestimated and the X-ray amorphous fraction is overestimated; for the same reason, DOC values of materials with different manganese contents, such as the as-received ore (42.4% [7]), are not directly comparable. The DOC values are therefore suitable for comparing samples within a series but not as the absolute fraction of the amorphous phase. In five independent calcinations under the same conditions, the hausmannite fraction of the crystalline phase determined semi-quantitatively by the reference intensity ratio (RIR) method ranged from 93.0% to 96.5% (mean 95.1%). Accordingly, the values above refer to crystalline hausmannite; the content of an X-ray amorphous component, which at the low Si content of the calcined ore (2.75%, Table 3) is predominantly manganese-bearing, cannot be determined by this method.

#### 3.2.2. Phase Composition of the Ash-Slag Waste

**Figure 2:**
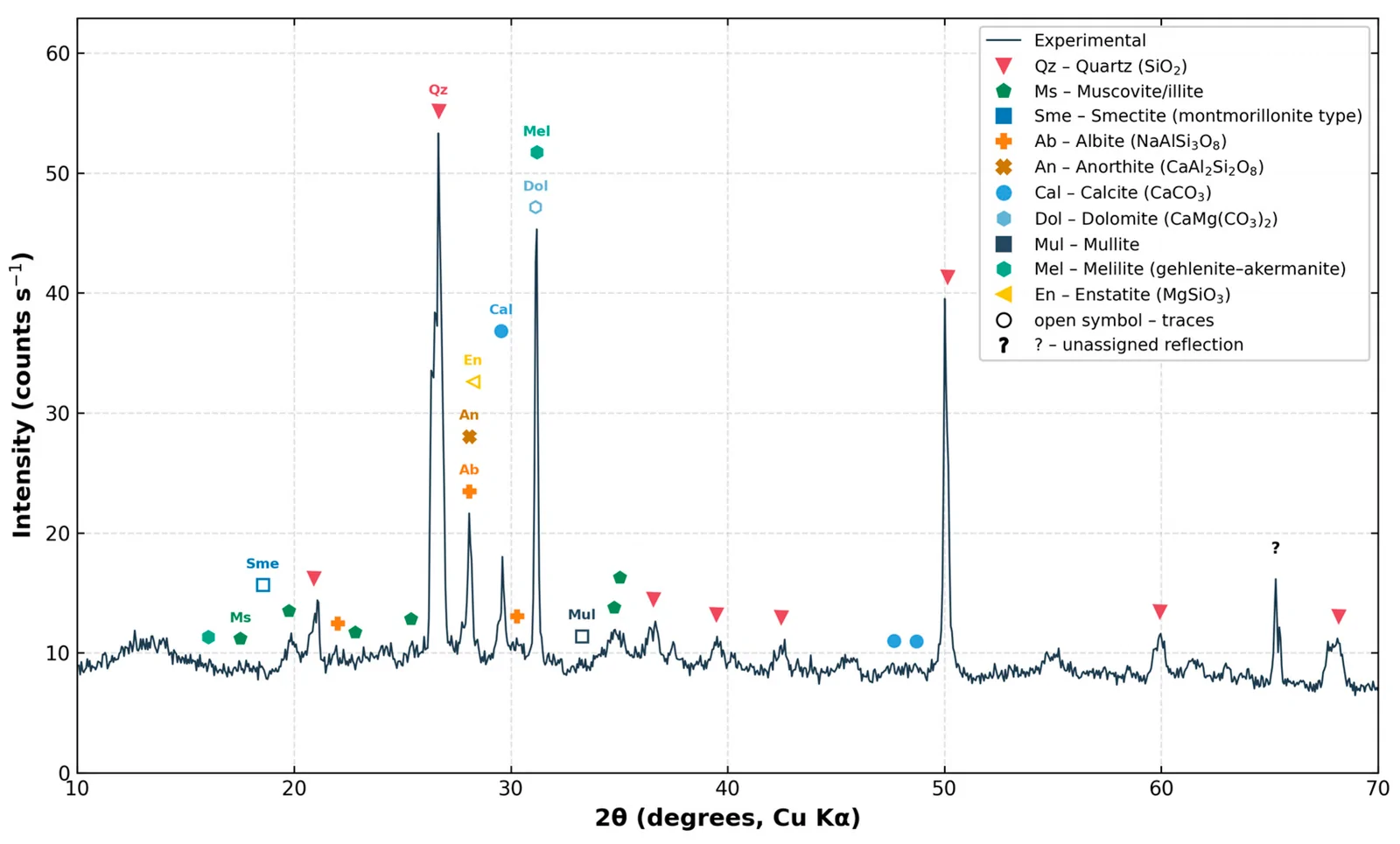
XRD pattern of the ash-slag waste from Pavlodar TPP-1 (Aluminium of Kazakhstan JSC). Filled symbols denote phases confirmed by Rietveld refinement and open symbols phases at trace level; phases without a reflection free of overlap are marked at their strongest overlapped reflection (ovl. in Table 6); ?—unassigned reflection.

XRD analysis of the ash-slag waste from Pavlodar TPP-1 (Figure 2) reveals a mineralogical composition that differs from the quartz–mullite–glass assemblage commonly reported for the combustion products of Ekibastuz-basin coal [4,14]. A weak amorphous halo of a glassy phase can be traced over the 2θ interval of 15–35°; since no internal standard was added, the amorphous fraction of the ASW was not quantified.

Rietveld refinement confirms α-quartz, muscovite/illite (1M_d_ and 2M_1_ polytypes), albite, anorthite, calcite and melilite (gehlenite–akermanite solid solution, Ca_2_(Al,Mg)(Si,Al)_2_O_7_) as the crystalline phases of the ASW, with smectite, dolomite, mullite and enstatite at trace level (Table 6). Smectite and dolomite cannot survive coal combustion; like part of the mica, they are attributed to secondary alteration of the ash in the disposal area (hydration and carbonation of the glass) and to admixed mineral matter, which is consistent with the relatively high K_2_O and MgO contents of the ASW (3.6% and 3.5%, Table 1). The dolomite contribution refines about 0.2° above the position of the dolomite 104 reflection (30.94°), with its lattice parameters at the limit of the tolerance, so this trace assignment is tentative, as in the clay (Section 3.2.3). Hematite, included in the initial model, did not exceed twice its estimated standard deviation and was removed, and magnetite, tested in a separate refinement, was not confirmed (its content refined to zero); the iron of the ASW (7.6% Fe_2_O_3_, Table 1) is presumably present mainly in the glassy phase and in the layer silicates. Mullite, which is commonly reported for Ekibastuz ash [4,14,15], improved the fit mainly near 26.0°, on the flank of the principal quartz reflection, and only weakly at its overlap-free reflection near 33.3° (Table 6), and is therefore reported only as a trace; gehlenite as a separate phase was not confirmed, since adding it to the model did not improve the fit at its characteristic reflections. The strongest melilite reflection (31.2°) is one of the most intense in the pattern (Figure 2); together with calcite, this indicates a notable contribution of calcium- and magnesium-bearing phases. On firing, calcite decomposes to CaO, which at 950 °C reacts mainly with the aluminosilicates to form calcium aluminosilicates such as anorthite and melilite [48,49]; melilite itself is a high-melting phase. Muscovite/illite is identified by its weaker reflections at 17.5, 25.4 and 34.8°, because its strongest basal reflection (about 8.8°) lies outside the scan range.

**Table 6:** Crystalline phases of the ash-slag waste (XRD, Rietveld refinement).

Phase	Status	Characteristic Reflections (2θ, °)
Quartz (Qz)	+	20.91, 26.66, 68.19
Muscovite/illite (Ms)	+	17.52, 19.76, 25.40, 34.75
Albite (Ab)	+	22.00, 28.08, 30.27
Anorthite (An)	+	28.08 (ovl.)
Calcite (Cal)	+	29.56, 47.67, 48.71
Melilite, gehlenite–akermanite (Mel)	+	16.04, 31.20
Smectite, montmorillonite type (Sme)	(+)	18.56
Dolomite (Dol)	(+)	31.14 (ovl.)
Mullite (Mul)	(+)	33.28
Enstatite (En)	(+)	28.24 (ovl.)

+ phase confirmed by Rietveld refinement; (+) phase at trace level, close to the detection limit. 2θ values are fitted positions corrected for sample displacement with quartz as an internal reference; ovl.—the phase has no reflection free of overlap, and its strongest overlapped reflection is given. Melilite denotes the gehlenite–akermanite solid solution; the dolomite assignment is tentative (Section 3.2.2). Mineral symbols follow the list of the International Mineralogical Association Commission on New Minerals, Nomenclature and Classification (IMA–CNMNC) [50].

The reflection at 2θ = 50.05° coincides with quartz 112, but its net intensity (71% of the principal quartz reflection) is several times that calculated for quartz in the refinement (9%), and a sharp reflection at 65.3° could not be assigned (question mark in Figure 2). Such isolated, excessively intense reflections are consistent with poor particle statistics of a few coarse crystallites; they were not used for phase identification.

#### 3.2.3. Phase Composition of the Clay Raw Material

**Figure 3:**
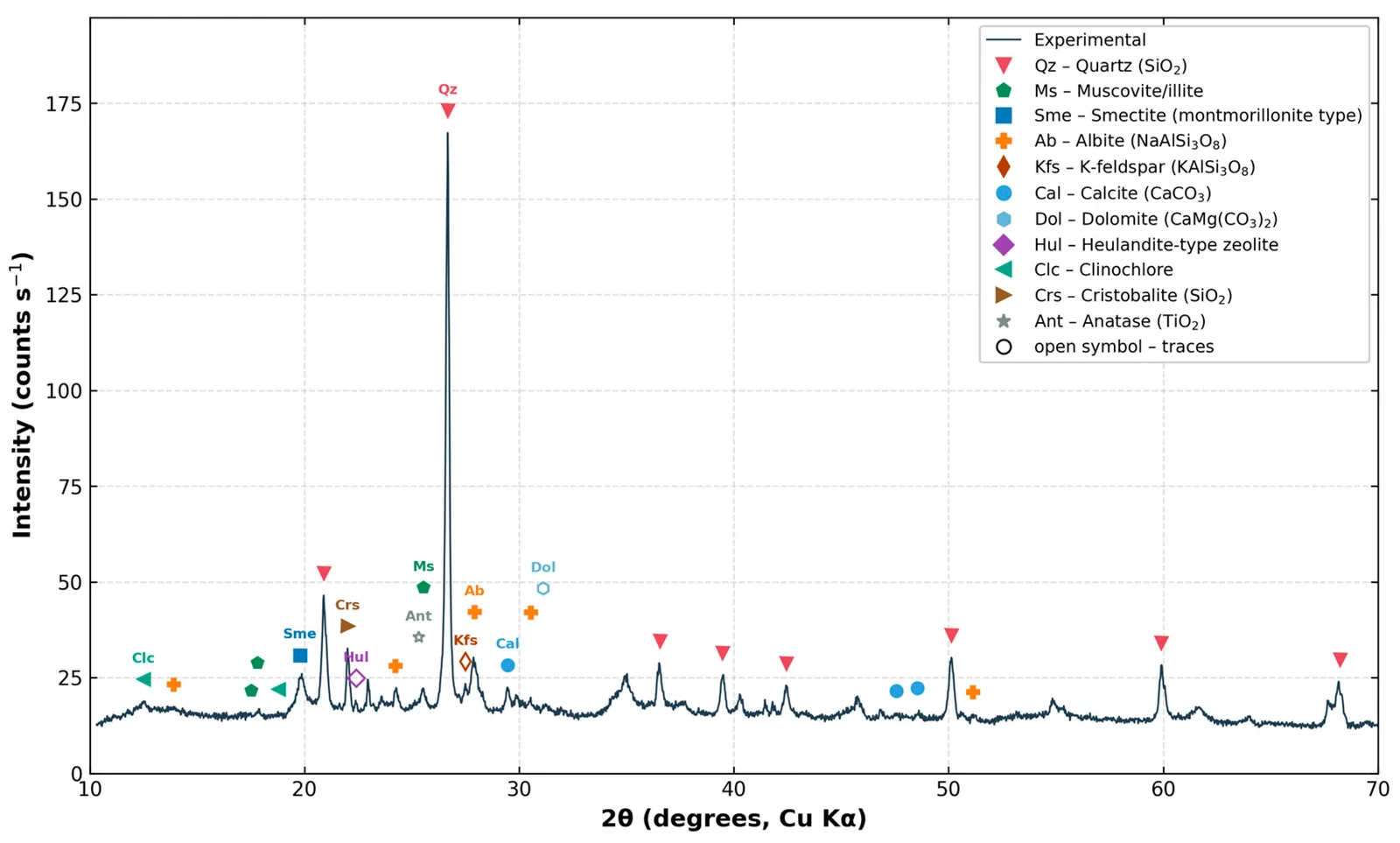
XRD pattern of the as-received clay used as the basic raw material. Symbols as in Figure 2.

Figure 3 shows the XRD pattern of the as-received clay. Rietveld refinement confirms quartz, muscovite/illite, a dioctahedral smectite of the montmorillonite type, albite, calcite, clinochlore and cristobalite, with dolomite, a heulandite-type zeolite, K-feldspar and anatase at trace level (Table 7). The smectite, recorded by its broad reflection at 19.8°, accounts for the medium plasticity of the raw material noted in Section 2.1, and calcite (29.5°) and dolomite are carriers of the CaO and MgO determined by XRF (Section 3.1). The dolomite contribution, however, refines about 0.2° above the position of the dolomite 104 reflection (30.94°), with its lattice parameters at the limit of the tolerance, so this trace assignment is tentative. The basal reflections of the smectite (about 6°) and of the zeolite (about 10°) lie below the scan range, so both were identified by their weaker reflections. Several phases suggested by automated search-match (vermiculite, stellerite, stilbite-Ca and an aluminotitanosilicate), as well as kaolinite, were not confirmed by the refinement. Reflections initially assigned to berlinite (AlPO_4_, PDF 00-010-0423) overlap with those of quartz, and phosphorus was not detected in the clay by XRF; this assignment was therefore rejected as an artifact.

#### 3.2.4. Phase Evolution of the Ceramic Mixes on Firing

**Figure 4:**
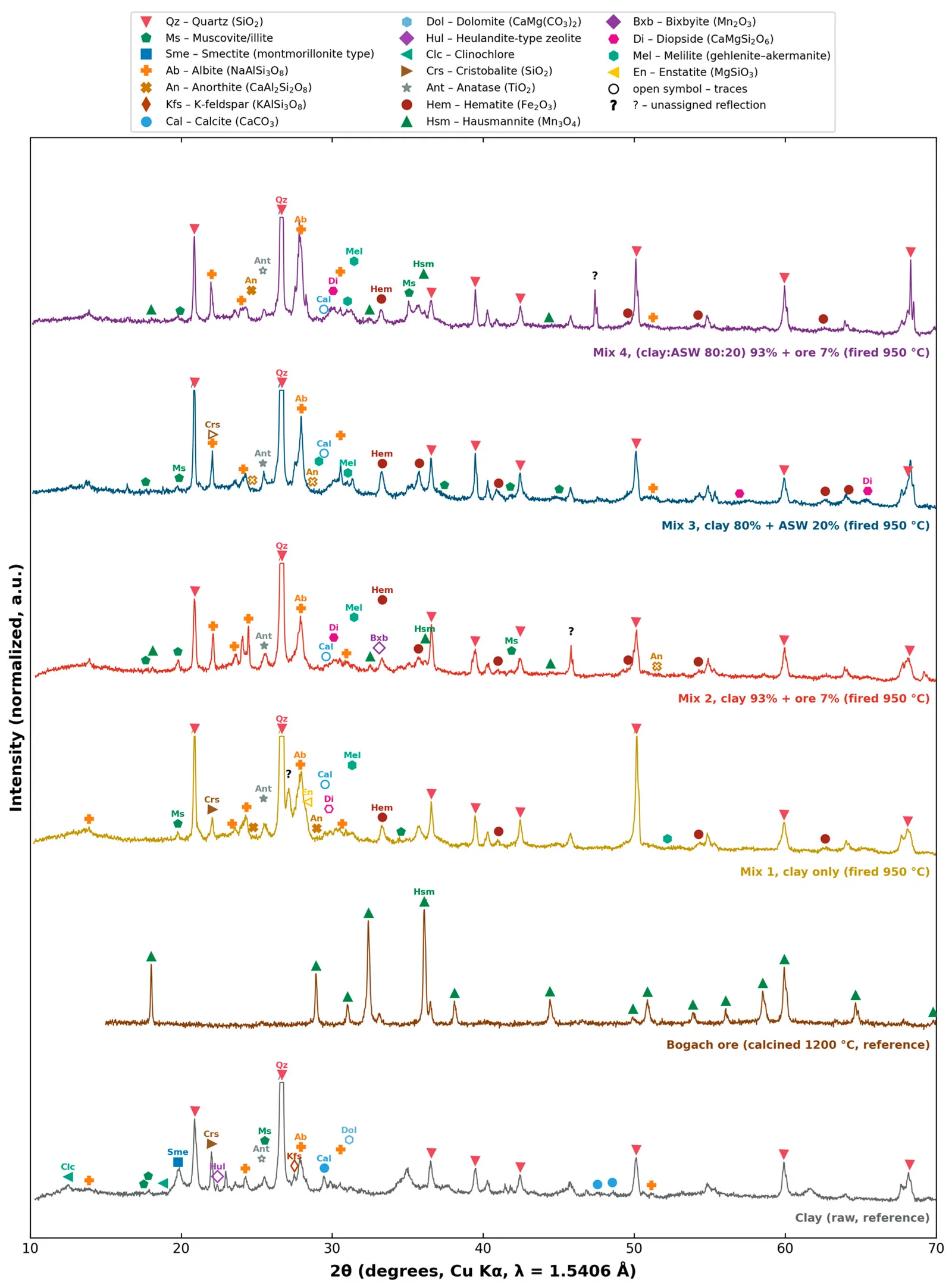
XRD patterns (bottom to top) of the as-received clay, the Bogach ore calcined at 1200 °C and Mixes 1–4 fired at 950 °C: Mix 1, clay only; Mix 2, clay 93% + ore 7%; Mix 3, clay 80% + ASW 20%; Mix 4, (clay:ASW = 80:20) 93% + ore 7%. Intensities are normalized by a common factor, and the strongest quartz reflections are truncated; the 2θ scale of each pattern is corrected for sample displacement using the quartz reflections. Only hausmannite is marked in the ore pattern. Symbols as in Figure 2.

To compare the mineralogical transformations that occur in the system clay–ASW–manganese ore on firing, the diffraction patterns of the as-received clay, the calcined ore and all four fired mixes are combined in Figure 4, and the phase composition is summarized in Table 8. The fired clay-only control (Mix 1) shows the changes produced by firing the clay itself: the reflections of smectite, clinochlore, the zeolite and dolomite disappear, calcite is retained only as a residual phase, and hematite (33.3° and 35.8°), anorthite, melilite and traces of diopside and enstatite appear, while quartz, albite, muscovite/illite, cristobalite and anatase persist. The formation of calcium–magnesium silicates from the decomposition products of the carbonates and the dehydroxylated clay minerals is characteristic of calcareous clays fired in this temperature range [35,48,49], and hematite accounts for the red-firing behavior of the clay. Essentially the same assemblage is recorded in Mixes 2–4; residual calcite is present at trace level in all fired mixes, cristobalite is retained as a trace in Mix 3 only, anatase is at trace level in Mix 4, and enstatite was found only as a trace in Mix 1 (Table 8). Calcite normally decomposes completely below about 900 °C [36], so its residual traces after firing at 950 °C may indicate locally lower temperatures in the kiln, which was not calibrated with pyrometric cones (Section 4), or partial recarbonation of free lime during storage of the fired specimens. Mullite was not confirmed in any fired mix (the trace mullite of the ASW, Table 6, diluted to 18.6–20% of the batch, is expected to fall below the detection limit), and K-feldspar was not detected after firing. Single sharp unassigned reflections in Mix 1 (27.1°), Mix 2 (45.8°) and Mix 4 (47.4°), marked with question marks in Figure 4, are attributed, as in the ASW, to isolated coarse crystallites.

In the ASW-containing Mixes 3 and 4, melilite is recorded by the reflection at 2θ ≈ 31.0°. Because melilite also forms in the ASW-free Mix 1, it cannot be attributed solely to the ASW, in which it is present before firing (Table 6). In Mixes 2 and 4, hausmannite is identified by its reflections at 2θ ≈ 18.0°, 32.4°, 36.1° and 44.4°, which deviate by no more than 0.12° from those of the calcined ore (Figure 4); Rietveld refinement gives 1.9 wt.% of the crystalline fraction in each mix (estimated standard deviation 0.1 wt.%; practical uncertainty about ±0.2 wt.%, see below). If all manganese of the ore were present as Mn_3_O_4_, the fired Mixes 2 and 4 would contain 4.2 and 4.3 wt.% Mn_3_O_4_; because the refined values refer to the crystalline fraction and are therefore upper bounds for the whole body, no more than about 46% of the manganese is present as crystalline hausmannite. The refined value is compatible with the crystalline hausmannite expected from the degree of crystallinity of the calcined ore (about 1.8 wt.% of the whole body, Section 3.2.1); the remainder of the manganese is attributed to the X-ray amorphous part of the ore and possibly to manganese dissolved in the glassy phase of the body. In the ore-free Mixes 1 and 3, these reflections are absent, and the refined values (0.2 wt.%) are not supported by observable reflections; values of this order lie below the practical detection limit and indicate the uncertainty of the values for Mixes 2 and 4. Hausmannite is therefore attributed to the manganese ore. When bixbyite (Mn_2_O_3_) and braunite were added to the final models, braunite was not detected in either mix, and bixbyite was not detected in Mix 4. In Mix 2, bixbyite refined to 1.0 ± 0.1 wt.% and the hausmannite content decreased to 1.4 wt.%; in this model, Mn_2_O_3_ would hold about 39% of the manganese present in crystalline oxides. This bixbyite content rests mainly on its strongest reflection near 32.9°, which lies on the low-angle flank of the hematite 104 reflection (33.3°), whereas its other reflections give only weak support. The bixbyite content is therefore probably overestimated by the overlap with hematite, and bixbyite is reported as a trace (Table 8). Partial oxidation of hausmannite to Mn_2_O_3_ on cooling nevertheless cannot be excluded for the ASW-free Mix 2; carry-over of Mn_2_O_3_ from the ore is less likely, because it would also be expected in Mix 4, which contains the same ore dosage. No evidence of phase transformations of crystalline hausmannite after firing at 950 °C was found in the ASW-containing Mix 4.

### 3.3. Processing and Physico-Mechanical Properties

The standard deviations of the bulk density and mass loss are small (relative standard deviations not exceeding 4.5% and 11%, respectively), so forming, drying and firing were reproducible. The linear shrinkage shows a larger scatter (for example, 2.35 ± 2.57% by height for Mix 1), because the dimensions were measured to the nearest 1 mm, which corresponds to about 2% of the specimen size. The total mass loss after firing (21.9% for Mixes 2–4 and 20.6% for Mix 1) is referred to as the green mass and therefore includes the moisture removed on drying. Referred to the dried mass, the mass loss on firing alone is 4.9% for Mix 3 and 6.1% for the control Mix 1; burnout of residual carbon from the ash-slag waste is not evident from these data. The complete set of processing and physico-mechanical properties is summarized in Table 9.

**Figure 5:**
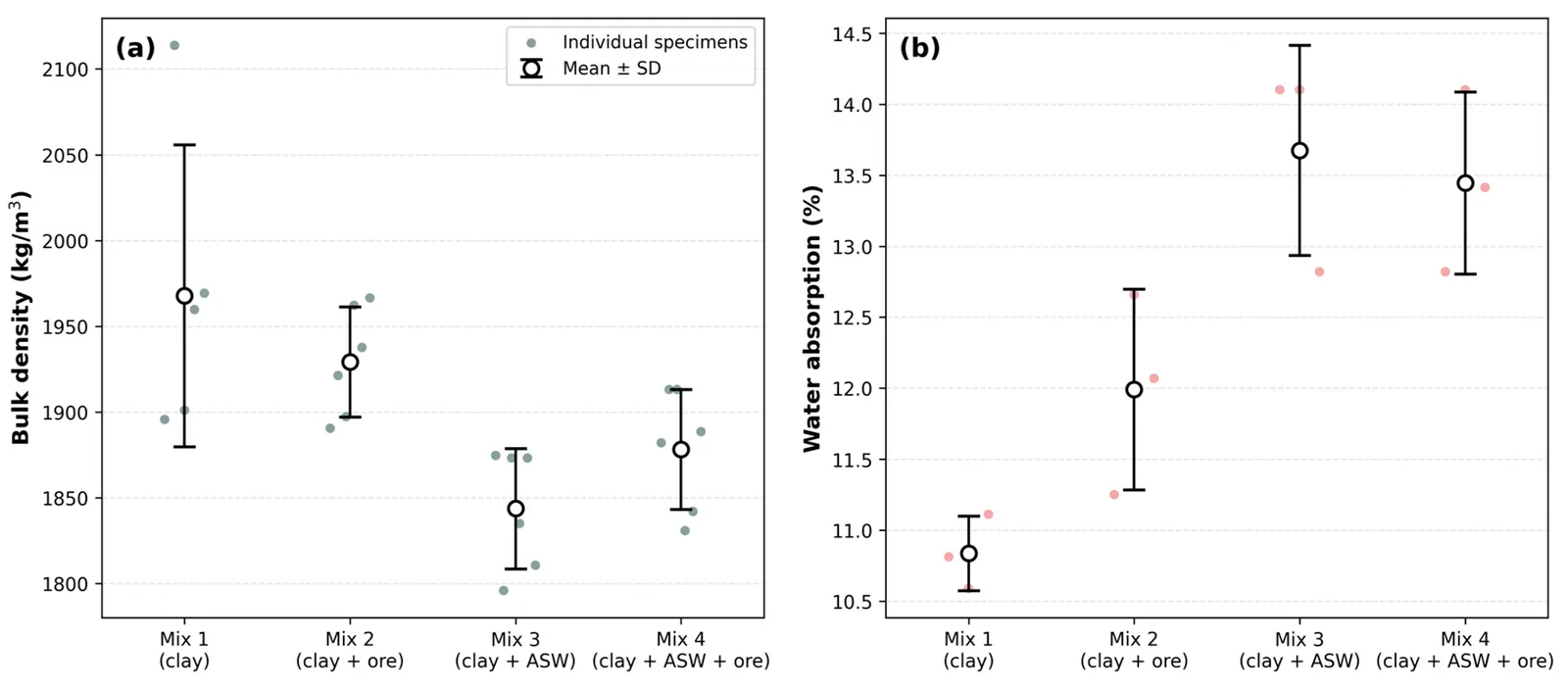
Bulk density (**a**) and water absorption (**b**) of the ceramic brick fired at 950 °C: open circles—mean ± standard deviation, dots—individual specimens (five to six per mix for density, three per mix for water absorption).

The variation in bulk density and water absorption with batch composition after firing at 950 °C is shown in Figure 5. The addition of 20% ASW reduces the density from 1968 to 1844 kg/m^3^ and raises the water absorption from 10.8% to 13.7%, which points to a higher open porosity. In Mix 4, the water absorption (13.4%) and density (1878 kg/m^3^) remain at the level of Mix 3; the differences are not statistically significant (Welch’s *t*-test, *p* = 0.71 and *p* = 0.12, respectively), and the water-absorption difference between them is below the weighing resolution (Section 2.4).

**Figure 6:**
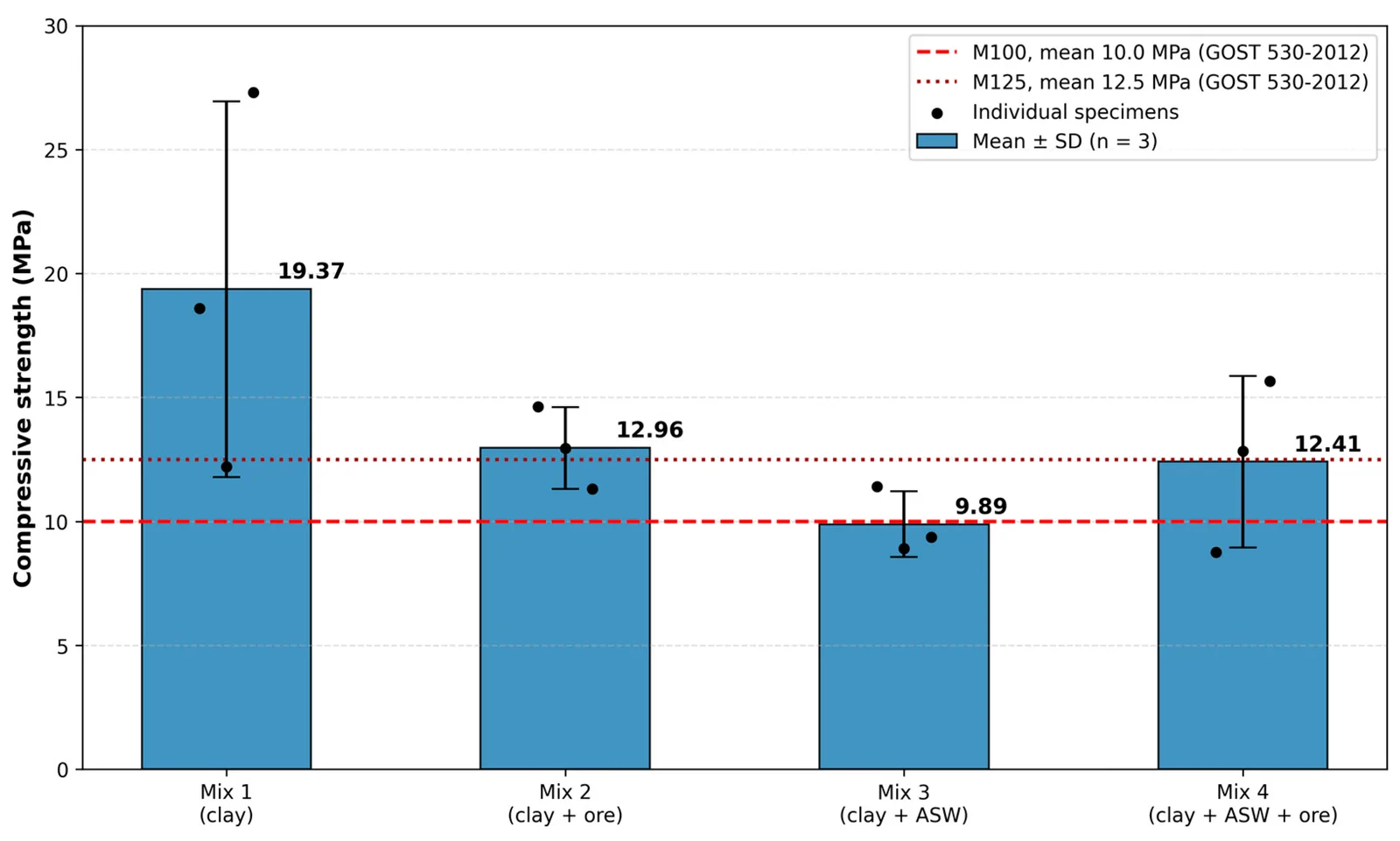
Compressive strength of the specimens studied (mean ± standard deviation, *n* = 3; points denote individual specimens). The dashed and dotted lines mark the minimum mean strengths for grades M100 and M125 according to [45].

The compressive strengths of the four mixes are shown in Figure 6. The scatter of the individual strength values is large for Mixes 1 and 4 (coefficients of variation 39% and 28%) and moderate for Mixes 2 and 3 (12.8% and 13.5%), whereas density and water absorption (Figure 5) vary much less (relative standard deviations up to 4.5% and 5.9%). Such scatter is common for small hand-molded specimens tested in small numbers and may reflect local differences in compaction and in the distribution of coarse particles. For practical application, it means that the grades in Section 3.4 describe the mean performance and carry a considerable uncertainty: the lowest individual value of Mix 4 (8.76 MPa) is only slightly above the individual minimum of ASTM grade NW (8.6 MPa), and the lowest value of Mix 1 limits its GOST grade to M125. Reliable grading requires tests on a larger number of standard-format units, and serial production would require control of batch homogenization and forming.

### 3.4. Compliance with Regulatory Requirements

The mixes were classified against three regulatory systems: the interstate standard GOST 530-2012 [45], used in the countries of the Commonwealth of Independent States; the European standard EN 771-1 [51] for clay masonry units; and the American standard ASTM C62-17 [52] for building brick. The summary classification is given in Table 10.

#### 3.4.1. GOST 530-2012

According to [45], the strength grade of brick is established from the mean compressive strength and the lowest individual value for five specimens, together with the flexural strength; the numerical value of the grade corresponds to the specified mean compressive strength in kgf/cm^2^ (≈0.098 MPa), and M100 is the lowest grade provided for solid and vertically perforated brick. Since the flexural strength was not determined and three specimens were tested, the grades below are estimates based on compressive strength. The mean strength of Mix 1 (19.37 MPa) corresponds to grade M175 (mean ≥ 17.5 MPa); however, its lowest individual value (12.2 MPa) is below the 15.0 MPa required for an individual specimen of this grade and the 12.5 MPa required for grade M150, so the full criterion gives grade M125. Mix 2 (12.96 MPa; lowest value 11.31 MPa) meets grade M125 (mean ≥ 12.5 MPa, individual ≥ 10.0 MPa), and Mix 4 (12.41 MPa; lowest value 8.76 MPa) meets grade M100 (mean ≥ 10.0 MPa, individual ≥ 7.5 MPa). The mean strength of Mix 3 (9.89 MPa) is below the 10.0 MPa required for grade M100, so this mix does not reach the lowest brick grade. The water absorption of all mixes (10.8–13.7%) satisfies the requirement of [45] that the water absorption of brick other than clinker brick be at least 6.0%.

The comparison between Mix 3 and Mix 4 shows that the addition of 7% calcined manganese ore to the clay–ASW system raised the mean strength from 9.89 to 12.41 MPa (a 25% gain); the ore-free Mix 3 does not reach grade M100, whereas Mix 4 does. With three specimens per mix and the large scatter of Mix 4 (8.76–15.65 MPa), this difference is not statistically significant (Welch’s *t*-test, *p* = 0.34) and should be regarded as a tendency that requires confirmation on a larger sample.

#### 3.4.2. EN 771-1:2011+A1:2015

The European standard [51] regulates the requirements for clay masonry units. Unlike GOST 530-2012, it does not impose a discrete strength scale but requires the manufacturer to declare the normalized mean strength f_b_ (in MPa) and, where required, the characteristic strength. For comparison with the other standards, the normalized strength is grouped here into classes of ≥5, ≥10, ≥15, ≥20 and ≥25 MPa; EN 771-1 and Eurocode 6 [53] use f_b_ as a continuous value. The normalized strength was calculated according to Annex A of EN 772-1 [47] as the product of the compressive strength of each specimen, the shape factor δ and the conditioning factor. The shape factor was obtained by linear interpolation of Table A.1 of EN 772-1 for the specimen height after firing (44–50 mm) and the smallest tabulated width (50 mm), since the specimen diameters (45–48 mm) are below it, which gives δ = 0.82–0.85. The specimens were stored under laboratory conditions after firing and were tested in the air-dry state, for which the conditioning factor is 1.0. Because the shape factors of EN 772-1 are defined for masonry units rather than for small cylindrical specimens, the resulting classes are indicative.

The mean normalized strengths are 16.1 MPa for Mix 1, 10.9 MPa for Mix 2, 8.3 MPa for Mix 3 and 10.4 MPa for Mix 4. On this basis, Mix 1 corresponds to the f_b_ ≥ 15 MPa class, Mixes 2 and 4 to the f_b_ ≥ 10 MPa class (for Mix 4 with a small margin), and Mix 3 to the f_b_ ≥ 5 MPa class.

The categories F0, F1 and F2 of EN 771-1 are not water-absorption classes: they designate the exposure conditions (freeze–thaw resistance) for which a masonry unit is declared, from F0 (passive exposure) to F2 (severe exposure), and they are established by freeze–thaw testing. Frost resistance was not determined in this work, so no F category is assigned to the mixes; the measured water absorption values (10.8–13.7%) are reported for information.

#### 3.4.3. ASTM C62-17

The standard [52] classifies solid fired-clay brick into three grades according to service conditions and frost-resistance requirements. Grade SW (severe weathering) is intended for structures exposed to intensive weathering and freezing and requires a mean compressive strength of at least 20.7 MPa (individual minimum 17.2 MPa) on the gross area at a maximum water absorption of 17% by the boiling method. Grade MW (moderate weathering) is designed for moderate climatic conditions and requires a minimum mean strength of 17.2 MPa (individual minimum 15.2 MPa) and water absorption of not more than 22%. Grade NW (negligible weathering) is intended for backup or interior masonry and requires a minimum mean strength of 10.3 MPa (individual minimum 8.6 MPa) with no water-absorption limit.

Mix 1 (19.37 MPa at 10.8% water absorption) exceeds the MW-grade mean requirement of 17.2 MPa; however, its lowest individual value (12.2 MPa) is below the MW individual minimum of 15.2 MPa, so Mix 1 formally meets grade NW, and an MW assignment would require additional testing on standard-format brick. Mix 2 (12.96 MPa; lowest value 11.31 MPa) and Mix 4 (12.41 MPa; lowest value 8.76 MPa) meet the NW-grade requirements (mean ≥ 10.3 MPa, individual minimum ≥ 8.6 MPa); for Mix 4 the margin of the individual minimum is small. Mix 3 (9.89 MPa) does not reach the mean requirement of grade NW (10.3 MPa). Since water absorption was determined in this work by 48 h immersion in water at 25 °C rather than by the 5 h boiling test of [52], the water-absorption limits of grades SW and MW were not applied; since three specimens were tested instead of the five required by [52], the ASTM grades are estimates based on compressive strength only.

For the mixes with a decorative function (Mixes 2 and 4), the standard ASTM C216 [54] for facing brick may also be considered, since it governs facing-brick requirements including appearance criteria. Its strength criteria are identical to those of ASTM C62-17 for grades SW and MW; the corresponding minimum values (17.2–20.7 MPa) exceed the results obtained for Mixes 2 and 4, which restricts their use as facing brick on the strength criterion alone. Their saturated dark-brown color is of interest for facing applications; however, frost resistance was not determined, and use under freeze–thaw exposure would require additional testing.

#### 3.4.4. Summary Classification

Mix 1, with the highest mean strength (19.37 MPa), corresponds to grade M175 per GOST 530-2012 and to the f_b_ ≥ 15 MPa class per EN 771-1 by the mean value; because of the high standard deviation (±7.58 MPa) and the low individual value of 12.2 MPa, the full criteria give grade M125 and ASTM C62-17 grade NW. Mix 2 (12.96 MPa) is classified as grade M125, class f_b_ ≥ 10 MPa and grade NW. Mix 3 (9.89 MPa) does not reach grade M100 or grade NW and corresponds to class f_b_ ≥ 5 MPa. Mix 4 (12.41 MPa) corresponds to grade M100, class f_b_ ≥ 10 MPa and grade NW. Three of the four mixes (Mixes 1, 2 and 4) meet the strength requirements of the lowest brick grades or classes in all three standards considered, whereas Mix 3 reaches only class f_b_ ≥ 5 MPa of EN 771-1; frost resistance was not assessed for any mix.

The highest mean strength is obtained for Mix 1 (19.37 MPa); however, the high standard deviation (±7.58 MPa, about 39% of the mean) reflects a large scatter between specimens. Mix 2 shows the lowest coefficient of variation (12.8%, compared with 13.5% for Mix 3) at grade M125, whereas the strength of Mix 4 varies over a wider range (8.76–15.65 MPa). From the perspective of the present study (developing mixes that incorporate industrial waste and provide a decorative effect), Mix 4 is of the greatest interest: it contains 20% ash-slag waste in the basic batch, reaches grade M100 by the mean strength (12.41 MPa, compared with 9.89 MPa for the ore-free Mix 3) and produces a saturated dark-brown body color. Mix 1, despite its higher mean strength, is the clay-only control without functional additives and contains no waste.

### 3.5. Correlation Analysis

**Figure 7:**
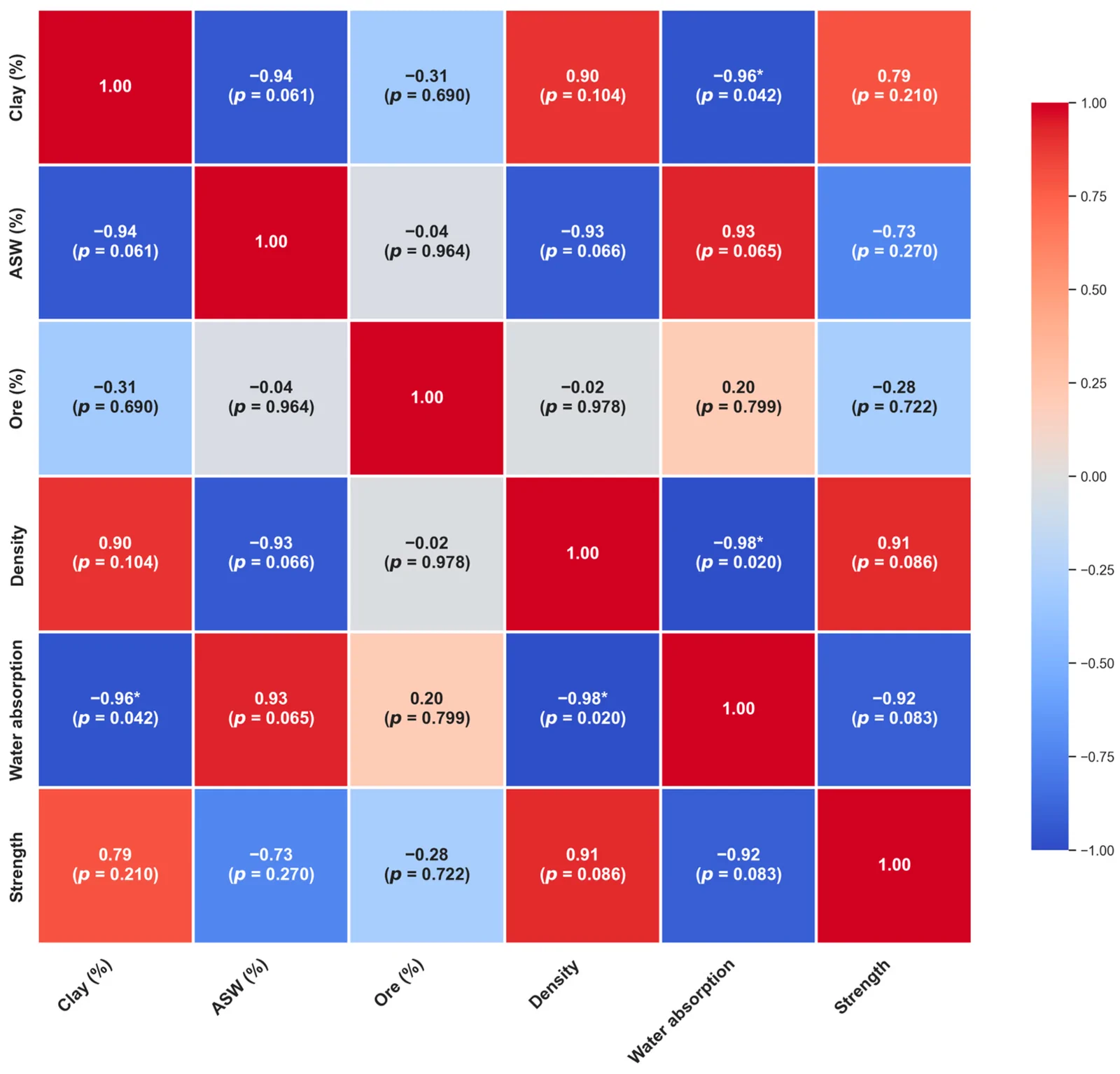
Correlation matrix relating batch composition (Table 4) to the properties of the ceramic brick (mean values of the four mixes). Significance level: * *p* < 0.05.

To quantify the observed relationships and the contribution of the batch components to the physico-mechanical characteristics, Pearson correlation coefficients (*r*) and the corresponding significance levels (*p*) were computed. The results are shown in Figure 7.

The clay acts as the principal structure-forming component. Clay content correlates positively with body density (*r* = 0.90, *p* = 0.10) and negatively with water absorption (*r* = −0.96, *p* = 0.042), consistent with a reduction in open porosity as the clay fraction increases; with four mixes, only the latter coefficient formally reaches the *p* < 0.05 threshold.

The ash-slag waste acts in the opposite direction: its content correlates positively with water absorption (*r* = 0.93, *p* = 0.065) and negatively with density (*r* = −0.93, *p* = 0.066), which is consistent with the lower density of the ash particles compared with clay. A trend of decreasing strength with increasing ash content (*r* = −0.73, *p* = 0.27) is also apparent.

Because the manganese-ore content had only two levels (0 and 7%) and its effect on strength differed in sign between the ASW-free and ASW-containing bodies, its correlations with the properties are weak (|*r*| ≤ 0.28, *p* ≥ 0.72) and do not characterize its effect; the influence of the ore can only be assessed from the direct comparison of Mix 4 with Mix 3 (Section 3.4.1).

Among the properties, density correlates negatively with water absorption (*r* = −0.98, *p* = 0.020) and positively with compressive strength (*r* = 0.91, *p* = 0.086), and strength correlates negatively with water absorption (*r* = −0.92, *p* = 0.083); of these, only the density–water absorption coefficient formally reaches the *p* < 0.05 threshold. These coefficients are computed over four between-mix mean values (two degrees of freedom) and describe the ranking of the mixes rather than an established quantitative dependence. They are consistent with the common role of open porosity in the density, water absorption and strength of the fired body, but they are not sufficient to use density as a criterion for property control without a larger data set. No correction for multiple comparisons was applied, and the composition variables are not independent (the clay content is fixed by the ASW and ore contents), so the coefficients are descriptive.

### 3.6. Decorative Properties

**Figure 8:**
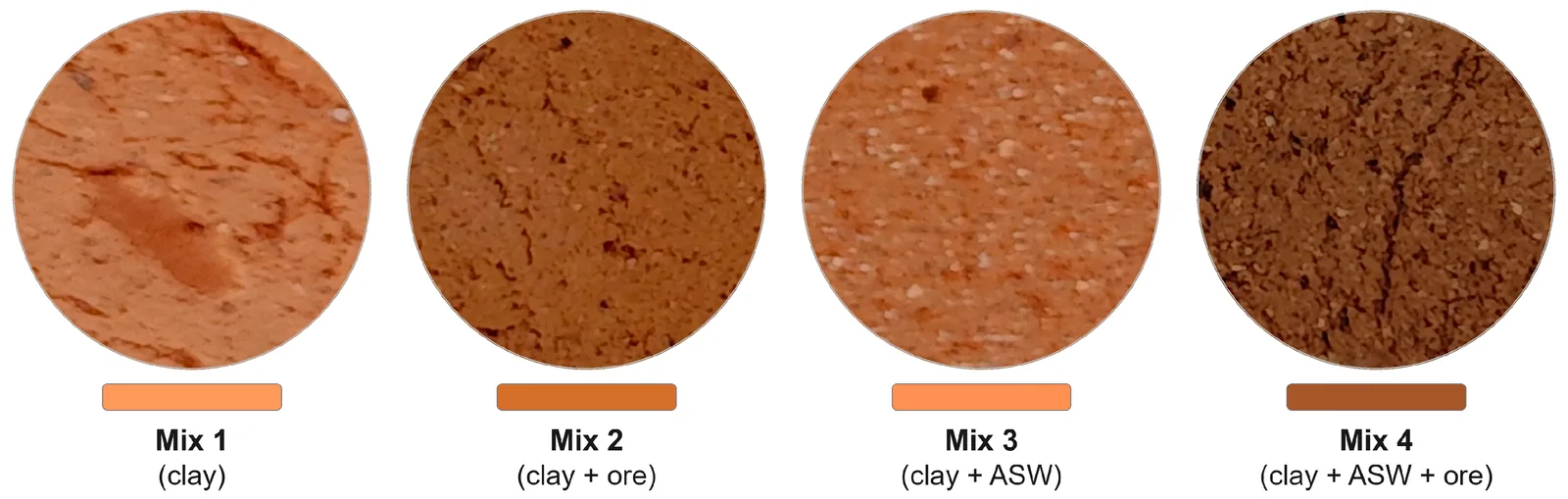
Visual appearance of the fired specimens of the four mixes; beneath each fragment, a patch of the mean color estimated from the image (sRGB equivalent of the CIELAB values in Table 11) is shown.

The visual appearance of the fired specimens is shown in Figure 8, and the corresponding colorimetric coordinates are given in Table 11.

The control (Mix 1) is light terracotta, with a high lightness (L* = 70.9) and a marked chroma (C* = 52.4). The introduction of 7% manganese ore (Mix 2) reduces L* from 70.9 to 54.5 (ΔE_00_ = 13.9) while chroma and hue angle remain practically unchanged; the body darkens without a hue shift. The addition of 20% ASW without ore (Mix 3) only slightly lowers the lightness (L* = 67.3; ΔE_00_ = 2.8) relative to the control and retains similar a*, b* and C* values.

The most pronounced change is observed for Mix 4 (clay–ASW blend with 7% ore), for which L* drops to 43.3 and chroma decreases to C* = 39.7. The total color difference relative to the control, ΔE_00_ = 25.6, is the largest in the series; in the CIE76 metric, the difference between Mix 4 and the control (ΔE*_ab_ = 30.3) far exceeds the value (ΔE*_ab_ ≈ 5) above which an observer perceives two different colors [55]. Relative to the ore-free Mix 3, the ore alone gives ΔE_00_ = 22.9. The hue angle of all four mixes lies within the narrow range of 57–60°, corresponding to the red-orange sector of the color circle, which shows that the calcined Bogach ore modifies mainly the lightness, and in the ASW-containing body also the chroma, with practically no hue shift.

The darkening is attributed to the manganese component introduced with the ore; hausmannite was detected by XRD in both Mixes 2 and 4 at similar contents (Section 3.2.4). Hausmannite absorbs strongly in the visible range [24], which is consistent with the transition from light terracotta to dark-brown tones and with the long use of manganese oxides as ceramic and glass colorants [18,19,20]. The colorimetric data quantify the darkening itself; its attribution to Mn_3_O_4_ rests on the XRD detection of hausmannite and on the optical properties of this phase reported in the literature [24], and it was not verified by optical measurements, such as diffuse reflectance spectroscopy, on the fired bodies. The stronger darkening of Mix 4 compared with Mix 2 at the same ore dosage was recorded on single specimens, and its cause has not been established. The Fe_2_O_3_ contents of the clay and the ASW are nearly equal (Table 1 and Table 5), so the ash does not supply additional iron; the higher measured Fe_2_O_3_ content of Mix 4 compared with Mix 2 (7.99% and 6.77%, Table 5), attributed to specimen heterogeneity (Section 3.1), may nevertheless have contributed. These data allow Mixes 2 and 4 to be considered as candidate materials for dark-toned decorative brick; use as facing brick would additionally require a higher strength (Section 3.4.3) and confirmed frost resistance.

## 4. Discussion

The introduction of 20% ash-slag waste (Mix 3) reduces the density from 1968 to 1844 kg/m^3^, raises the water absorption from 10.8% to 13.7% and reduces the mean compressive strength from 19.37 to 9.89 MPa, below the lower bound of grade M100. This strength loss (about 49%) is comparable to the reduction of more than 50% reported for 20% fly ash in bricks fired at about 800 °C [10] and larger than the 22% reported for 20% bottom ash at 900 °C [9]. For the fly-ash bricks, a firing temperature of about 1050 °C was considered necessary to reach higher strength [10]. The loss observed here is consistent with the moderate firing temperature and with the weak amorphous halo of this ASW (Section 3.2.2), which suggests a limited content of reactive glass. This effect is attributed mainly to dilution of the clay matrix by the ash components (Section 3.2.2 and Table 6): quartz, feldspars and melilite are likely to take little part in sintering at 950 °C, muscovite/illite may contribute as a flux, and CaO from calcite is bound mainly in calcium silicates and aluminosilicates at this temperature [48,49]. A contribution of the burnout of residual carbon, a common source of open porosity in ash-bearing bodies, is not evident from the mass loss on firing (Section 3.3). The values obtained lie within the broad range reported for fired clay bricks with coal ash: bricks with up to 20% fly ash fired at about 800 °C in an industrial kiln met a local minimum of 8.25 MPa [10], whereas bodies with bottom ash fired at 1100–1150 °C exceeded 20 MPa [9]; in both cases the water absorption increased with the ash share [8,9,10].

The introduction of 7% calcined Bogach manganese ore (Mix 4) raises the mean compressive strength to 12.41 MPa, a 25% gain over Mix 3, which corresponds to a transition to grade M100, while water absorption (13.4%) and density (1878 kg/m^3^) remain at the level of Mix 3. With three specimens per mix, the strength difference is not statistically significant (*p* = 0.34), and the effect of the ore on strength should be regarded as a tendency. At the same time, the ore addition did not lower the mean strength of the ASW-containing body. In the ASW-free system, by contrast, the ore addition was accompanied by a lower mean strength (12.96 MPa for Mix 2 versus 19.37 MPa for Mix 1), a lower density (1929 versus 1968 kg/m^3^) and a higher water absorption (12.0% versus 10.8%), although the latter difference does not exceed the weighing resolution (Section 2.4). Because of the large scatter of Mix 1, this strength difference is not statistically significant (Welch’s *t*-test, *p* = 0.28), and a two-way analysis of variance of the individual values does not confirm an interaction between the ore and ASW factors (*p* = 0.11). The opposite signs of the two differences only suggest that the effect of the ore on strength may depend on the presence of ASW in the batch; the behavior of the ASW-free body is consistent with the strength reduction reported for a manganese-containing pigment in ceramic brick [22].

A possible explanation of the strength tendency involves the pre-formed hausmannite phase introduced with the ore calcined at 1200 °C. Pre-calcination is an essential step of the formulation studied here: it converts the manganese minerals of the ore into hausmannite before the ore enters the batch, so that the brick firing at 950 °C does not have to accomplish this transformation. The recrystallization of the matrix during firing (formation of hematite and melilite, together with anorthite and diopside, partly at trace level) occurs in all mixes, including the clay-only control (Section 3.2.4), and therefore does not by itself explain the difference between Mixes 3 and 4. According to XRD, Mixes 2 and 4 display the hausmannite reflections at 2θ ≈ 18.0°, 32.4°, 36.1° and 44.4°, corresponding to the tetragonal lattice I4_1_/amd and matching the reflections of the calcined Bogach ore (Section 3.2.1); manganese silicates were not detected, and Mn_2_O_3_ was found only as a possible trace in the ASW-free Mix 2 (Section 3.2.4). The persistence of crystalline Mn_3_O_4_ at the brick-firing temperature is therefore supported for both ore-containing mixes and is consistent with the stability of hausmannite in air above approximately 890 °C [26,27]. Below this temperature, Mn_3_O_4_ is metastable in air with respect to Mn_2_O_3_, so its persistence after cooling reflects slow oxidation kinetics, which depend on crystallite size [33,34]; a trace of Mn_2_O_3_ in Mix 2 would be consistent with such partial oxidation. The crystalline fraction of the calcined ore contains 95.77% hausmannite with an average coherent-scattering domain size of 113.4 ± 4.5 nm [7]; this value characterizes the crystallites rather than the particles of the ground product. It is hypothesized that the Mn_3_O_4_ particles act as heterogeneous nucleation centers for the newly forming silicate phases. Such a mechanism would also have to explain why no strength gain was observed in the ASW-free Mix 2; it was not verified by microstructural observation, and no direct data on the strengthening mechanism were obtained in the present work.

The effect of manganese-bearing additives on the strength of clay ceramics differs between systems: manganese-containing wastes did not lower the strength of colored brick [23], whereas a manganese-containing pigment reduced it [22]; the use of manganese ores beyond metallurgical processing, including silicate materials, has been discussed [7]. In the present data, the density and water absorption of Mix 4 do not differ significantly from those of Mix 3 (Section 3.3), so a fluxing contribution of the ore to densification is not confirmed.

The decorative effect is associated with the manganese component of the batch. The ore lowers the lightness in the ASW-free body (L* from 70.9 to 54.5) and, more strongly, in the ASW-containing body (from 67.3 to 43.3); this is qualitatively consistent with the integral absorption of Mn_3_O_4_ in the visible range, which arises from LMCT transitions O^2−^ → Mn^3+^ in the 410–585 nm region [24]. The confinement of the hue angle to the narrow 57–60° interval indicates that the ore addition lowers the lightness without shifting the hue; the chroma is unchanged in the ASW-free body (C* 52.4 and 52.6) and decreases in the ASW-containing body (from 51.5 to 39.7). At 900–1000 °C, manganese oxides may also react with hematite to form iron–manganese spinels such as jacobsite [19], which may contribute to the darkening.

By mean compressive strength, the resulting mix complies with three regulatory systems: grade M100 per [45], class f_b_ ≥ 10 MPa per [51] and grade NW per [52] (Table 10). Frost resistance was not determined, so no EN 771-1 exposure category (F0–F2) can be assigned, and use under freeze–thaw cycling requires additional testing. The mix takes up 20% ash-slag waste from Pavlodar TPP-1 in the basic batch and uses Bogach manganese ore as a colorant, an application outside conventional metallurgical processing [7].

Several limitations of the present study should be acknowledged. The experiments were carried out on laboratory-formed cylindrical specimens with a single ore dosage (7%) and a single firing temperature (950 °C). The results therefore characterize this formulation and do not establish an optimum ore content or firing schedule. Direct extrapolation to the industrial production of standard-format brick is also limited: larger units fired in industrial kilns develop temperature gradients and follow longer firing cycles, which may change both the phase development and the uniformity of the color. The firing temperature corresponds to the reading of the kiln controller; the kiln was not calibrated with pyrometric cones. Since all mixes were fired in the same kiln under the same program, this does not affect the comparison between mixes. Water absorption and compressive strength were determined on three specimens per mix without rejection of individual results, and at this sample size the strength difference between Mix 4 and Mix 3 is not statistically significant; the correlation analysis in Section 3.5 was performed over only four between-mix mean values. The normalized strength was obtained by applying the shape factors of EN 772-1, which are defined for masonry units, to small cylindrical specimens, so the EN 771-1 classes are indicative. The color coordinates were measured on one specimen per mix from a single photograph. The manganese ore used here had been calcined at 1200 °C beforehand, so the hausmannite phase was introduced into the batch in a ready form; whether a comparable effect can be achieved with as-received ore, relying on its phase transformation during brick firing, was not investigated. With as-received ore, the cryptomelane, braunite and calcite of the ore [7] would decompose and transform within the body during firing, and the release of CO_2_ from the carbonate would add porosity; both the strength and the color may therefore differ from those obtained with the calcined ore. In addition, inside the brick body the access of oxygen is restricted and may be further reduced by the burnout of organic matter and by the CO_2_ released from carbonates, so that the manganese oxides of as-received ore could be reduced further than on calcination in air and bound as Mn^2+^ in silicates or in the glassy phase, which would also change the color. The calcination step also adds an energy cost that has to be included in any economic assessment. The high strength scatter of the control Mix 1 (standard deviation ±7.58 MPa, about 39% of the mean) calls for a larger sample size before a grade can be assigned reliably. Finally, flexural strength, frost resistance, long-term strength and weathering of the body were not evaluated, which leaves open the question of compliance with the exposure categories of [51] and grades MW and SW [52].

These limitations define the directions for further research. Priority tasks include compressive- and flexural-strength testing on at least five specimens per mix and the assessment of frost resistance in accordance with [44] and the corresponding European and American test methods, to establish the exposure categories of [51] and grades MW and SW [52]; long-term weathering trials on standard-format industrial units are also required. The manganese-ore content should be varied over the 5–15% range and the firing temperature over the 950–1100 °C range to optimize the strength and decorative characteristics, and comparative tests with as-received manganese ore would quantify the contribution of the calcination step. Finally, a techno-economic and environmental assessment of the full cycle of ore mining, calcination at 1200 °C and brick production is needed to evaluate the commercial feasibility of the technology.

## 5. Conclusions

This work investigated the effect of a 7% addition of calcined Bogach manganese ore and Pavlodar TPP-1 ash-slag waste (20% of the clay–ASW blend) on the properties of ceramic brick fired at 950 °C. The bodies were examined by X-ray fluorescence, X-ray diffraction with Rietveld refinement, CIELAB colorimetry (CIEDE2000) and compressive-strength, water-absorption and shrinkage tests and were classified against GOST 530-2012, EN 771-1 and ASTM C62-17. The principal results are as follows.

1. A ceramic brick based on Mix 4 (93% of a clay:ASW = 80:20 blend with 7% calcined Bogach manganese ore, i.e., 74.4% clay, 18.6% ASW and 7.0% ore) was obtained with a mean compressive strength of 12.41 ± 3.46 MPa (three specimens), corresponding by the mean value to grade M100 per [45], class f_b_ ≥ 10 MPa per [51] and grade NW per [52].
2. The introduction of 7% calcined manganese ore into the clay–ASW system raised the mean strength by 25% relative to the ore-free batch (from 9.89 to 12.41 MPa); with three specimens per mix, this difference is not statistically significant (*p* = 0.34). Water absorption (13.4% versus 13.7%) and bulk density (1878 versus 1844 kg/m^3^) remained at the level of the ore-free batch. In the ASW-free body, the ore addition was accompanied by a lower mean strength (12.96 versus 19.37 MPa; *p* = 0.28).
3. XRD shows that hausmannite (Mn_3_O_4_), introduced with the ore calcined at 1200 °C, persists as a crystalline phase after firing at 950 °C in both ore-containing mixes (Mixes 2 and 4) and is absent from the ore-free mixes; only a possible trace of Mn_2_O_3_ was found in the ASW-free Mix 2. Firing of the clay produces hematite and melilite in all mixes, including the clay-only control, together with anorthite and diopside (partly at trace level), whereas mullite was not confirmed. The mechanism of the strength change has not been established.
4. Image-based CIELAB colorimetry shows a decrease in lightness from L* = 70.9 to 43.3 for Mix 4 and the largest total color difference relative to the control in the series (ΔE_00_ = 25.6; 22.9 relative to the ore-free Mix 3), with the hue angle preserved within the 57–60° range. This makes the mix a candidate material for dark-brown decorative brick, subject to frost-resistance testing.
5. Across the four mixes, density correlates with water absorption (*r* = −0.98, *p* = 0.020), whereas its correlation with compressive strength (*r* = 0.91, *p* = 0.086) does not reach formal significance; with four mean values, these relationships describe the ranking of the mixes and require confirmation on a larger data set.
6. Ash-slag waste from Pavlodar TPP-1 (18.6% of the total batch mass) and calcined Bogach manganese ore (7%) can thus be used together in decorative construction ceramics.

The principal scientific contribution of this work is the characterization of a clay–ASW body containing a pre-formed hausmannite phase introduced with a calcined manganese ore: the ore darkens the body at an essentially constant hue without lowering the mean strength of the ASW-containing body, and crystalline hausmannite persists in the fired aluminosilicate matrix at 950 °C. A possible mechanism of the strength tendency, involving heterogeneous nucleation of silicate phases on Mn_3_O_4_ particles, requires verification by microstructural studies. In applied terms, the results suggest a joint use of Central Kazakhstan manganese ores and Pavlodar coal-ash waste in construction ceramics that meet the regional, European and American strength requirements for ordinary brick of the lowest grades.

These conclusions refer to laboratory specimens with one ore dosage (7%) fired at one temperature (950 °C), with three specimens per mix for strength. Before industrial use, the formulation requires pilot-scale firing of standard-format units, frost-resistance and durability testing, a comparison with as-received ore and a techno-economic assessment that includes the calcination of the ore.

## Figures and Tables

**Table 1:** Chemical composition of the ash-slag waste from Pavlodar TPP-1 (XRF, normalized to 100%, loss on ignition not included, wt.%).

SiO_2_	Al_2_O_3_	Fe_2_O_3_	CaO	MgO	K_2_O	TiO_2_	Na_2_O	P_2_O_5_	SO_3_	MnO
61.1	18.6	7.6	3.8	3.5	3.6	1.0	0.1	0.2	0.4	0.1

**Table 2:** Particle-size distribution of the ash-slag waste from Pavlodar TPP-1.

Fraction (mm)	Content (%)
>2.5	3.9
2.5–1.6	1.2
1.6–1.0	1.6
1.0–0.63	2.3
0.63–0.315	2.7
0.315–0.16	46.5
<0.16 (passing)	41.8

**Table 3:** Elemental composition of the Bogach manganese ore in the as-received state and after calcination at 1200 °C (XRF, standardless analysis without normalization to 100%, wt.%).

*T* (°C)	Mn	Ca	Si	Ba	K	Sr	Fe	Mg	Al	Pb	Zn	Na	Ti
25	60.77	5.44	3.49	2.05	2.04	1.42	0.89	1.00	0.82	0.21	0.09	0.04	0.05
1200	65.24	4.45	2.75	1.82	1.59	1.51	0.95	0.76	0.58	0.18	0.11	0.02	0.03

**Table 4:** Compositions of the ceramic mixes investigated.

Mix	Clay (%)	ASW (%)	Ore (%)	Description
1	100.0	0	0	Control (clay only)
2	93.0	0	7.0	Clay + 7.0% ore
3	80.0	20.0	0	Clay:ASW = 80:20
4	74.4	18.6	7.0	(Clay:ASW = 80:20) + 7.0% ore

ASW—ash-slag waste. Compositions are expressed in wt.% relative to the total batch mass; the ore was introduced after calcination at 1200 °C.

**Table 5:** Oxide composition of the ceramic mixes (XRF of fired specimens, normalized to 100%, wt.%).

Oxide	Mix 1 (Clay)	Mix 2 (Clay + Ore)	Mix 3 (Clay + ASW)	Mix 4 (Clay + ASW + Ore)
SiO_2_	62.03	58.00	60.19	56.87
Al_2_O_3_	17.89	17.26	19.00	18.71
Fe_2_O_3_	7.54	6.77	9.06	7.99
CaO	3.22	4.12	3.23	3.79
MgO	3.33	3.51	3.08	3.33
K_2_O	2.74	2.84	2.46	2.27
SO_3_	1.37	1.68	1.20	1.18
TiO_2_	0.77	0.72	0.83	0.70
MnO	0.07	3.87	0.14	4.02
BaO	0.11	0.21	0.08	0.22
Na_2_O	<0.01	0.08	0.10	0.07
Other	0.93	0.94	0.63	0.85

ASW—ash-slag waste. Data are normalized to 100%; loss on ignition (LOI) is not included. The RSD for the major oxides does not exceed 0.6%; <0.01—below 0.01%.

**Table 7:** Crystalline phases of the as-received clay (XRD, Rietveld refinement).

Phase	Status	Characteristic Reflections (2θ, °)
Quartz (Qz)	+	20.90, 26.67, 50.13
Muscovite/illite (Ms)	+	17.52, 25.54
Smectite, montmorillonite type (Sme)	+	19.79
Albite (Ab)	+	24.23, 27.92, 30.54
Calcite (Cal)	+	29.47, 47.58, 48.55
Clinochlore (Clc)	+	12.50, 18.79
Cristobalite (Crs)	+	22.01
K-feldspar (Kfs)	(+)	27.50
Dolomite (Dol)	(+)	31.12 (ovl.)
Heulandite-type zeolite (Hul)	(+)	22.41 (ovl.)
Anatase (Ant)	(+)	25.32 (ovl.)

Symbols and notation as in Table 6; the dolomite assignment is tentative (Section 3.2.2 and Section 3.2.3).

**Table 8:** Phase composition of the clay and of the ceramic mixes fired at 950 °C (XRD, Rietveld refinement).

Phase	Clay (Raw)	Mix 1	Mix 2	Mix 3	Mix 4
Quartz (Qz)	+	+	+	+	+
Muscovite/illite (Ms)	+	+	+	+	+
Smectite, montmorillonite type (Sme)	+	−	−	−	−
Albite (Ab)	+	+	+	+	+
Anorthite (An)	−	+	(+)	(+)	+
K-feldspar (Kfs)	(+)	−	−	−	−
Calcite (Cal)	+	(+)	(+)	(+)	(+)
Dolomite (Dol)	(+)	−	−	−	−
Heulandite-type zeolite (Hul)	(+)	−	−	−	−
Clinochlore (Clc)	+	−	−	−	−
Cristobalite (Crs)	+	+	−	(+)	−
Anatase (Ant)	(+)	+	+	+	(+)
Hematite (Hem)	−	+	+	+	+
Hausmannite, Mn_3_O_4_ (Hsm)	−	−	+	−	+
Bixbyite, Mn_2_O_3_ (Bxb)	−	−	(+)	−	−
Diopside (Di)	−	(+)	+	+	+
Melilite, gehlenite–akermanite (Mel)	−	+	+	+	+
Enstatite (En)	−	(+)	−	−	−

Symbols as in Table 6; − not detected. Bixbyite was tested in a separate refinement with Mn_2_O_3_ and braunite added to the final models (Section 3.2.4). Mix 1 is the fired clay-only control; the column “Clay (raw)” refers to the unfired clay.

**Table 9:** Properties of the mixes studied after firing at 950 °C.

Mix	Bulk Density (kg/m^3^)	Water Absorption WA (%)	Linear Shrinkage L_d_ (%)	Linear Shrinkage L_h_ (%)	Mass Loss W_dry_ (%)	Mass Loss W_fired_ (%)	Compressive Strength (MPa)
1	1968 ± 88	10.8 ± 0.3	6.44 ± 1.64	2.35 ± 2.57	15.42 ± 1.66	20.55 ± 1.33	19.37 ± 7.58
2	1929 ± 32	12.0 ± 0.7	5.24 ± 0.80	5.14 ± 1.08	17.77 ± 0.54	21.87 ± 0.62	12.96 ± 1.66
3	1844 ± 35	13.7 ± 0.7	2.91 ± 1.35	4.93 ± 1.08	17.89 ± 0.27	21.90 ± 0.23	9.89 ± 1.33
4	1878 ± 35	13.4 ± 0.6	3.65 ± 1.66	3.81 ± 0.49	17.59 ± 0.40	21.87 ± 0.42	12.41 ± 3.46

Values are given as mean ± standard deviation: bulk density over five (Mix 1) or six (Mixes 2–4) specimens; linear shrinkage and mass loss over five (Mixes 1 and 2) or six (Mix 3) specimens, and for Mix 4 over five (linear shrinkage) or six (mass loss) specimens; water absorption and compressive strength over three specimens per mix. L_d_, L_h_, W_dry_ and W_fired_ as defined by Equations (1)–(4). Statistics in the text and in the correlation analysis (Section 3.5) were computed from the unrounded values.

**Table 10:** Classification of the mixes studied against GOST 530-2012, EN 771-1:2011+A1:2015 and ASTM C62-17.

Mix	Compressive Strength (MPa)	Water Absorption (%)	GOST 530-2012	EN 771-1 f_b_, MPa (Class)	ASTM C62-17
1	19.37 ± 7.58	10.8 ± 0.3	M125 ^1^	16.1 (≥15)	NW ^1^
2	12.96 ± 1.66	12.0 ± 0.7	M125	10.9 (≥10)	NW
3	9.89 ± 1.33	13.7 ± 0.7	below M100	8.3 (≥5)	below NW ^2^
4	12.41 ± 3.46	13.4 ± 0.6	M100	10.4 (≥10)	NW

^1^ By the mean value alone, Mix 1 would meet grade M175 and the MW mean requirement (17.2 MPa); its lowest individual value (12.2 MPa) is below the individual minima of grades M175 (15.0 MPa) and M150 (12.5 MPa) and of grade MW (15.2 MPa). ^2^ The mean (9.89 MPa) is below the mean requirement of grade NW (10.3 MPa). GOST grades are estimated from compressive strength only; ASTM grades are assigned by compressive strength only (water absorption was not determined by the 5 h boiling test); f_b_—mean normalized compressive strength according to EN 772-1, Annex A (δ = 0.82–0.85, conditioning factor 1.0), indicative for cylindrical specimens; frost resistance (EN 771-1 categories F0–F2) was not determined.

**Table 11:** CIELAB color coordinates of the fired specimens (image-based estimate; sRGB color space, D65 white point).

Mix	L*	a*	b*	C*	h° (°)	ΔE_00_
1 (clay)	70.9	28.5	44.0	52.4	57.1	–
2 (clay + ore)	54.5	26.8	45.3	52.6	59.4	13.9
3 (clay + ASW)	67.3	27.5	43.6	51.5	57.8	2.8
4 (clay + ASW + ore)	43.3	21.4	33.4	39.7	57.4	25.6

L*—lightness; a*, b*—chromatic coordinates; C*—chroma; h°—hue angle; ΔE_00_—total color difference relative to the clay-only control, Mix 1 (CIEDE2000). Color differences were computed from the unrounded coordinates; recalculation from the rounded values in the table may differ by 0.1.

## Data Availability

The original contributions presented in this study are included in the article. Further inquiries can be directed to the corresponding authors.

## Abbreviations

The following abbreviations are used in this manuscript:

ASW	Ash-slag waste
CALPHAD	Calculation of phase diagrams
CIE	Commission Internationale de l’Éclairage
CIELAB	CIE 1976 L*a*b* color space
CIEDE2000	CIE 2000 color-difference formula
COD	Crystallography Open Database
DOC	Degree of crystallinity
ICDD	International Centre for Diffraction Data
IMA–CNMNC	International Mineralogical Association Commission on New Minerals, Nomenclature and Classification
JPEG	Joint Photographic Experts Group (image format)
JSC	Joint-stock company
LMCT	Ligand-to-metal charge transfer
LOI	Loss on ignition
PDF	Powder Diffraction File
PI	Plasticity index
RIR	Reference intensity ratio
RSD	Relative standard deviation
R_wp_	Weighted-profile R-factor
SD	Standard deviation
sRGB	Standard RGB color space
TPP	Thermal power plant
XRD	X-ray diffraction
XRF	X-ray fluorescence
